# The role of verbal and pictorial information in multimodal incidental acquisition of foreign language vocabulary

**DOI:** 10.1080/17470218.2014.979211

**Published:** 2015-07-01

**Authors:** Marie-Josée Bisson, Walter J. B. van Heuven, Kathy Conklin, Richard J. Tunney

**Affiliations:** 1 School of Psychology, Nottingham University, Nottingham, UK; 2 School of English, Nottingham University, Nottingham, UK

**Keywords:** Incidental learning, Vocabulary acquisition, Eye tracking, Multimodalities, Foreign language learning

## Abstract

This study used eye tracking to investigate the allocation of attention to multimodal stimuli during an incidental learning situation, as well as its impact on subsequent explicit learning. Participants were exposed to foreign language (FL) auditory words on their own, in conjunction with written native language (NL) translations, or with both written NL translations and pictures. Incidental acquisition of FL words was assessed the following day through an explicit learning task where participants learned to recognize translation equivalents, as well as one week later through recall and translation recognition tests. Results showed higher accuracy scores in the explicit learning task for FL words presented with meaning during incidental learning, whether written meaning or both written meaning and picture, than for FL words presented auditorily only. However, participants recalled significantly more FL words after a week delay if they had been presented with a picture during incidental learning. In addition, the time spent looking at the pictures during incidental learning significantly predicted recognition and recall scores one week later. Overall, results demonstrated the impact of exposure to multimodal stimuli on subsequent explicit learning, as well as the important role that pictorial information can play in incidental vocabulary acquisition.

There are many benefits to learning other languages such as an awareness and appreciation of other cultures, as well as metalinguistic and cognitive benefits (e.g., Bialystok, 2008; Sanz, 2013; Yelland, Pollard, & Mercuri, 1993). In addition, in a global economy, being able to speak another language increases one's chances of finding employment (see Graddol, 2006). Although language learning is important and desirable, many find it a difficult and frustrating experience as there are many words to learn. One way of facilitating vocabulary learning is through incidental learning situations. In this type of situation, vocabulary learning occurs through mere exposure to foreign language (FL) input whilst learners are engaged in a variety of tasks, following which a surprise vocabulary test is normally administered (Horst, 2005; Hulstijn, 2001; Laufer, 2001).

Much research on incidental FL vocabulary acquisition has taken place in the context of reading (e.g., Brown, Waring, & Donkaewbua, 2008; Horst, 2005; Hulstijn, Hollander, & Greidanus, 1996; Kweon & Kim, 2008; Pellicer-Sánchez & Schmitt, 2010; Pigada & Schmitt, 2006; Rott, 1999; Vidal, 2011; Waring & Takaki, 2003), listening (Brown et al., 2008; Vidal, 2011), and reading-while-listening (Brown et al., 2008; Horst, Cobb, & Meara, 1998; Webb, Newton, & Chang, 2013). These studies highlight the potential of these types of incidental learning situations for promoting incidental FL vocabulary acquisition. It is puzzling, however, that so much more research has been conducted on the acquisition of FL vocabulary through a reading situation that might favour the learning of orthographic word form and orthographic form–meaning links. In contrast, there is much less research on listening, which might have had a positive impact on the incidental learning of FL phonological word forms and form–meaning links, or reading-while-listening, which promotes both the learning of phonological and orthographic word forms and form–meaning links. The research that has been done indicates that incidental learning from spoken input appears to be more difficult for learners (see Brown et al., 2008; Vidal, 2011). However, providing the orthographic forms of the words seems to facilitate learning by allowing learners to segment auditory word forms into more manageable chunks. Could learning also be facilitated by using other combinations of information?

In the above studies, learners could only derive the meaning of the new words from the context of the sentence they were reading and/or listening to. Another way of facilitating meaning acquisition for learners is to provide some additional input in their native language (NL). Lambert, Boehler, and Sidoti (1981) and Lambert and Holobow (1984) used combinations of languages while students read and/or listened to FL radio programmes. Some students had access to both spoken and written input in the FL (which is similar to the reading-while-listening studies mentioned earlier) whilst other students received some of the input in their NL either through the soundtrack or in writing. Although these studies focused on listening comprehension, the first study revealed that participants were better at understanding the meaning of words in context when they had received the FL in writing (Lambert et al., 1981), whilst the second study indicated that having the NL in the spoken input and FL in writing led to better contextual meaning comprehension (Lambert & Holobow, 1984). Taking the results of both studies together, Lambert and Holobow (1984) concluded that the best input for learners was having the NL spoken dialogue combined with written FL script. Unfortunately, this work with dual language input in the context of radio programmes was discontinued.

The studies mentioned so far all included verbal information that was presented through written and/or auditory modality and sometimes included a combination of languages in order for learners to extract meaning more easily. Another way of facilitating meaning acquisition of new FL words is to expose learners to FL input in combination with pictorial information. One advantage of using pictorial information is that even complete beginners can access word meaning, as they can derive the meaning of FL words from the pictures.

Previous research has investigated the incidental acquisition of FL vocabulary through watching films with subtitles. This is a situation with auditory verbal information in the film's soundtrack, written verbal information in the subtitles, and pictorial information in the films’ dynamic images. In recent years, FL films with subtitles have become increasingly popular and are easily accessible on the Internet. Furthermore, even when watching a NL film, subtitles in many languages are often available at the click of a button, and therefore FL input can easily be added to many NL films. Language researchers quickly became interested in this multimodal situation as a potential source of incidental vocabulary acquisition, since a combination of FL and NL can be used in conjunction with pictorial information thereby providing an information-rich situation. Depending on the type of subtitles, it is possible to have the FL in the soundtrack and NL in the subtitles (standard subtitling), FL in the subtitles and NL in the soundtrack (reversed subtitles), or FL in both (intralingual subtitles). Intralingual subtitling in the NL, sometimes called captioning, is also available during many television programmes to make them accessible to the hard of hearing and deaf community.

Early work on the effectiveness of using films with subtitles to promote incidental FL vocabulary acquisition was conducted by d'Ydewalle and Pavakanun (1995). In two experiments, participants (adults in Experiment 1 and children in Experiment 2) watched a 12-minute cartoon, following which they completed a 5-alternative forced-choice (AFC) meaning recognition vocabulary test. This study was unique as it included all possible subtitling conditions as well as many possible control conditions (e.g., FL soundtrack with no subtitles, NL soundtrack with no subtitles, no soundtrack with FL subtitles, etc.). The disadvantage of the design was that the number of participants in each condition was small (fewer than 10 per condition). The authors concluded that the adult data showed evidence of vocabulary acquisition, with the groups of participants with standard and reversed subtitles performing best. However, the results of their group with reversed and standard subtitles did not differ significantly from the results of a group who was exposed to intralingual subtitles in the NL, suggesting that the results of the vocabulary tests might not be due to the learning of FL vocabulary. Furthermore, in their second experiment with children, they found no significant differences between the groups on the vocabulary test scores. In a similar further study, d'Ydewalle and Pavakanun (1997) concluded once more that considerable vocabulary acquisition occurred from watching a short subtitled video and that reversed subtitles enhanced vocabulary acquisition more than standard subtitles. Unfortunately, no statistical analyses were provided to support their conclusions. In another study, d'Ydewalle and van de Poel (1999) focused on standard and reversed subtitles only. They used a 10-minute still-motion movie with Danish or French as a FL, following which participants completed a 10-item auditory and a 10-item 3-AFC meaning recognition test. For Danish as FL, a significant increase in written test performance was found in both standard and reversed subtitle groups compared to a control group with NL intralingual subtitles. In the auditory test, only the standard subtitles group performed better than the control group. However, no significant French vocabulary acquisition was found. The authors therefore concluded that incidental vocabulary acquisition might be facilitated when the FL and the NL are similar (participants in this study were native speakers of Dutch, which is more similar to Danish than French). Their results with Danish as a FL, however, would have been more convincing had the vocabulary tests included more items. In fact, the significant differences between the groups amount to about one more word being correctly recognized.

A similar study by Koolstra and Beentjes (1999) involved more items on the vocabulary test (28 items). The results of this study also showed a small increment in vocabulary acquisition with children correctly recognizing the meaning of two more words if they had watched a FL video with NL subtitles (standard subtitles) compared to a control group who watched a different movie, and only one more word when compared to group who watched the FL video with no subtitles. These results need to be interpreted with caution, however, as within-subject analyses were conducted despite the design being between subjects. Taken together, the results of these studies suggest that incidental vocabulary acquisition from watching films with subtitles is possible. However, in view of the limitations of many of these studies, more research is warranted.

Gullberg, Roberts, and Dimroth (2012) investigated the incidental acquisition of FL words using a multimodal situation with FL auditory information and pictorial information. More specifically, they asked participants to watch a short weather report in a FL, following which they measured word form recognition. The results revealed that participants were able to recognize 57.5% of the auditorily presented target words as having occurred in the weather report, indicating early incidental learning of word form (percentage accuracy for target items calculated from Table 2 of Gullberg et al., 2012).

Incidental learning of form–meaning links was observed in another study using a multimodal situation (Bisson, van Heuven, Conklin, & Tunney, 2013). In this study, participants were presented with FL word forms, both auditorily and in writing, as well as line drawings depicting the meaning of the words. As this was done in the context of a letter-search task, only the written FL word was relevant to the task. However, through the presentation of the line drawings, participants could link the FL word forms to meaning representations. Following the incidental learning task, participants were asked to explicitly learn FL words through a translation recognition task where they were presented with auditory FL word forms along with possible written NL translations equivalent. Their task was to indicate whether the written NL words were the correct translations for the auditory FL word forms, and they received feedback on their answers to allow them to learn the correct translations. Unbeknownst to participants, half of the words in the explicit learning task had occurred during the incidental learning situation. The results showed higher accuracy scores in the explicit learning task for the words presented during the incidental learning task. This incidental learning advantage was found immediately after the incidental learning situation, the next day, and one week later (Bisson et al., 2013).

In a follow-up study, Bisson, van Heuven, Conklin, and Tunney (2014b) varied the number of exposures (2, 4, 6, and 8) to the FL words during the letter-search task. The results showed that as little as two exposures to words in the incidental learning task was sufficient for incidental vocabulary acquisition to occur. Furthermore, the authors found that the initial exposures had more of an impact on incidental learning than the subsequent exposures. This might have been due to the fact that although the pictures were not relevant to the task, participants might look at them at first (novelty effect). However, towards the end of the experiment they may have been less interested in the pictures.

In the two studies from Bisson et al. (2013, 2014b). FL acquisition occurred when both the auditory and written word forms presented during the incidental learning phase were in the FL. Therefore in order to derive meaning from the FL word forms, participants had to process the pictures. This situation is similar to an intralingual subtitle situation, in which both the soundtrack and subtitles are presented in the FL. The advantage of this type of situation is that even complete beginners can benefit from it, as the pictures provide meaning information, and therefore the meaning of the FL words does not have to be derived from the context, as in, for example, reading. However, in a multimodal situation, such as a film with subtitles, it is also possible to provide meaning information in one of the verbal streams—that is, through either the audio or the written information. In fact, most subtitled films are presented with FL soundtrack and NL subtitles (standard subtitling). With standard subtitles, one can enjoy the visual aspects of the film and the original soundtrack and understand the story through the NL subtitles. Although it is clear that people read NL subtitles (Bisson, van Heuven, Conklin, & Tunney, 2014a; d'Ydewalle & de Bruycker, 2007), it is less certain whether the FL words in the soundtrack are also processed. Since the subtitles provide a NL translation of the dialogue, there is no need to attend to the FL words. However, in order to learn FL vocabulary from watching a FL film with NL subtitles, it is essential that the FL word forms in the soundtrack are processed. As mentioned earlier, a few incidental learning studies using FL films with NL subtitles concluded that learning occurred (d'Ydewalle & Pavakanun, 1995, 1997; d'Ydewalle & van de Poel, 1999; Koolstra & Beentjes, 1999), which suggests that the FL words in the soundtrack were processed.

A landmark study by Saffran, Newport, Aslin, Tunick, and Barrueco (1997), which has become known as the Saffran task, also provided evidence for the processing of irrelevant auditory information during an incidental learning task. In this study, participants were exposed to a continuous recording containing six pseudowords made from syllables from an artificial language while they completed an unrelated computer task (creating computer illustrations). Following the exposure phase, participants had to complete a surprise 2-AFC word form recognition test using the pseudowords from the recording, as well as new pseudowords made up of the same syllables as foils. The results showed that both adults and children correctly chose words from the tape with 59% accuracy. A further group of participants repeated the task on two consecutive days and achieved 73% (adults) and 68% (children) accuracy. This study illustrated that both adults and children are able to use statistical information to extract words from a continuous speech stream and that this process can happen incidentally while attention is focused elsewhere. In this study, however, participants could only extract word form information as there was no meaning attached to these words. Furthermore, the auditory information consisted of only six nonwords repeated 300 times, and therefore it is difficult to compare this situation to a film soundtrack where most words are not repeated so much.

In the multimodal situation used in Bisson et al. (2013, 2014b), words were repeated from 2 to 8 times only, and this was sufficient for FL vocabulary acquisition to occur. Participants were able to recognize the correct translation equivalent of the FL auditory words in a test phase, suggesting that even irrelevant auditory information was processed during an incidental learning phase. However, because written FL word forms were also provided, it is also possible that this was responsible for the learning, or at least contributed to it, especially since the letter-search task required participants to search the written FL word. Participants may have linked both the written and auditory FL word forms to the meaning representation accessed from the pictures during the letter-search task. Having had access to both written and auditory FL word forms may have facilitated learning (see Bird & Williams, 2002; Hu, 2008; Ricketts, Bishop, & Nation, 2009; Rosenthal & Ehri, 2008). In contrast, it is less clear to what extent FL auditory word forms were processed and how much they contributed to the learning effect. The first aim of the current study was therefore to assess the incidental acquisition of FL vocabulary using a situation similar to a film with standard subtitles. The simple multimodal situation used in Bisson et al. (2013, 2014b) was used; however, in the present study, written NL translations of the FL auditory word forms were provided instead of FL written words. Therefore, any learning that occurs can only be explained by the processing of FL auditory word forms.

Another interesting question, which arises from using a situation similar to standard subtitling, is whether there is an added benefit of having access to pictures in addition to written translations. If participants can access the meaning of the FL words through the NL translations, the meaning information provided by the pictures becomes redundant. Furthermore, the pictures are not necessary to complete the letter-search task, whilst the written translations are. Previous work on free recall of information predicts that having access to a picture during encoding will be beneficial for recall (picture superiority effect), as a picture can be encoded both as verbal and as nonverbal information (Paivio & Csapo, 1973). This dual-coding theory suggests that both verbal and nonverbal information can then serve as a cue at retrieval (Paivio & Csapo, 1973). Furthermore, Nelson, Reed, and McEvoy (1977) suggested that this picture superiority effect is due to the distinctiveness of pictorial information and that pictures are better remembered because they benefit from a more direct connection to semantic representations (sensory–semantic model). Although little is known about the impact of pictorial information in an incidental learning paradigm involving a FL, the picture superiority effect predicts that the use of multimodal (visual and verbal) input will benefit learning.

In the field of FL learning, the findings with regards to picture superiority effects are not as clear as those obtained in memory research. For instance, Lotto and de Groot (1998) found no superiority effect for pictures in explicit learning of FL words. In fact, their results showed better FL word learning when their participants were presented with FL and NL word pairs during a learning phase than when they were presented with FL words in combination with pictures. Similarly, Carpenter and Olson (2011) found no advantage for using pictures and FL word pairs during an explicit learning phase, which they explained by participant's overconfidence in their ability to recall FL words from pictures. Once they eliminated this bias, however, a picture superiority effect did emerge. The second aim of the current study was therefore to assess the impact of pictorial information on the incidental acquisition of FL vocabulary.

In order to address this question, a similar letter-search task was used to provide an incidental learning situation as in Bisson et al. (2013, 2014b). However, the type of information presented for each FL word varied within participants. Participants were presented with three different types of trials: auditory FL word forms only, auditory FL word forms with written NL translations, and both auditory FL word forms and written NL translations presented with simple line drawings depicting the meaning of the words (see Figure 1). In Bisson et al. (2013, 2014b), incidental vocabulary acquisition was assessed through an explicit learning task using translation recognition with feedback. In the present study, the same task was used to assess learning the day following the incidental learning phase. Participants also returned one week later to complete a recall test and a translation recognition test (without feedback). It was predicted that having access to all three types of information (auditory FL word forms, written NL translations, and pictures) would be beneficial for learning as assessed by accuracy scores on the explicit learning task the next day, as well as on the recall and recognition tests one week later. Furthermore, the accuracy scores on both word conditions presented with meaning, whether written translations or both written translations and pictures, should be higher than those for the words presented with auditory FL word forms only. Furthermore, a control group who did not take part in the incidental learning phase completed both the explicit learning task and the delayed recall and recognition tests. It was predicted that the experimental group would outperform the control group for words presented with meaning in the incidental learning phase.

**Figure 1.**
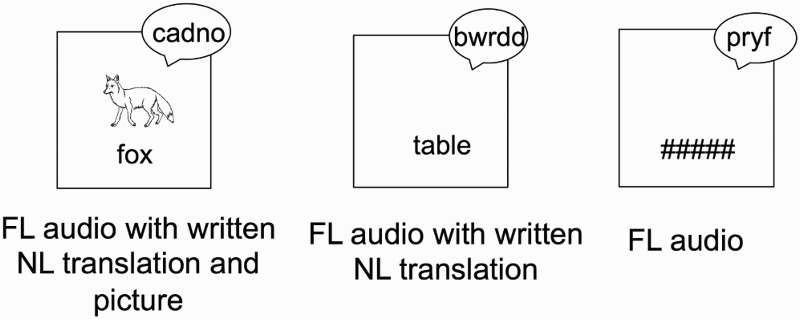
Example of the three types of word presentations during the incidental learning phase (letter-search task). FL = foreign language; NL = native language.

As was explained earlier, because the current experiment presented the written information in the NL, it was possible to assess whether the irrelevant FL auditory word forms were processed, as this was the only way participants could learn the FL word form. However, as meaning information was presented through both NL translations and pictorial information, the learning of FL word meaning could occur whether either or both were processed. Thus, the study used eye tracking to assess the allocation of attention to the different elements of the multimodal situation in order to assess its impact on vocabulary acquisition. It was predicted that participants would spend a considerable amount of time looking at the written word because of the letter-search task. However, the processing of the pictures was less certain because they were irrelevant for the letter-search task. In order to increase the likelihood that participants would look at the pictures, these were presented slightly earlier than the onset of the written words (300 ms). This timing of the presentation of the different elements of the multimodal situation also mimicked a film with subtitles: Film viewers are normally looking at the images in a film when subtitles appear on screen. It was important that participants looked at the pictures since the eye-tracking data will serve to investigate the impact of the pictorial information on the learning of the FL words. Based on the picture superiority effect, it was expected that the time spent looking at the pictures would predict scores on the explicit learning task and the recall and recognition test. It was also expected that the time spent looking at the NL word would predict the learning outcomes.

Finally, as it was found in Bisson et al. (2014b) that the first few exposures to FL words and pictures led to bigger learning gains than subsequent exposures, the eye-tracking data were also used to investigate the allocation of attention to the different elements of the multimodal situation *across the duration of the incidental learning situation*. The incidental learning phase was therefore split into six blocks of trials for the purpose of the analysis. The prediction was that the time spent looking at the pictures would decrease across the duration of the incidental learning phase. In contrast, as the written words were necessary to complete the letter-search task, it was predicted that participants’ viewing behaviour would be more constant throughout the experiment.

## Experimental Study

### Method

#### Participants

Sixty-six students from the University of Nottingham took part in the experiment and received course credit or payment for their participation. All participants completed a self-reporting language questionnaire at the end of the experiment to ensure that they were native English speakers and that they had no prior knowledge of the FL (Welsh) used in the experiment. Ten participants were excluded on the basis that they were either non-native speakers of English (four participants) or they had prior knowledge of Welsh or a related language (six participants). A further two participants were excluded from the analyses as they were unable to complete all parts of the experiment, and one further participant was excluded because of technical difficulties with the eye-tracker. The final sample therefore included 28 participants in the experimental group (mean age = 23.1 years, 21 females) and 25 participants in the control group (mean age = 22.8 years, 18 females).

#### Design

A mixed design was used in this experiment with group as a between-subject factor (2 levels: control group and experimental group) and word condition during the incidental learning phase (3 levels: auditory FL word form only, A; auditory FL word form and written NL translation, AW; auditory FL word form and written NL translation with a picture illustrating the meaning, AWP) as a within-subject factor.

#### Stimuli

The stimuli consisted of 78 auditory Welsh words and their written English translations and pictures illustrating the meaning of the words (from Snodgrass & Vanderwart, 1980). Three lists of stimuli including 26 words from each word condition (A, AW, and AWP) were created to allow for counterbalancing across participants. For example, Participant 1 was assigned to List 1 where 26 words were presented auditorily (A), 26 words were presented auditorily with their NL translations (AW), and 26 words were presented auditorily along with their NL translation and pictures (AWP). Participant 2 was assigned to List 2, Participant 3 to List 3, Participant 4 to List 1, and so on. The 26 words in each word condition were counterbalanced across the lists, such that, for example, the words presented in the A condition in List 1 were presented in the AW condition in List 2, and in the AWP condition in List 3. Each participant in the control group was also assigned to one of the three stimuli lists prior to taking part in the experiment in order to collate their accuracy scores according to the three word conditions for the purpose of the analyses. The lists of stimuli were counterbalanced across participants in both the experimental and the control groups.

#### Procedure

There were three phases to this experiment: Phase 1 was the incidental learning phase, Phase 2 was the explicit learning phase, and Phase 3 was the recall and recognition phase. The experimental group completed all the phases, whilst the control group started with Phase 2. Phase 1 was completed on the first day of the experiment, Phase 2 the next day, and, finally, Phase 3 one week later.

##### Phase 1: Incidental learning

In Phase 1 of the experiment, participants in the experimental group completed a letter-search task whilst their eye movements were recorded with an Eyelink 1000 (SR Research, Canada) desktop eye-tracker (sampling rate of 1000 Hz). A chin rest was used to immobilize the participant's head.

The procedure was similar to the one used in Bisson et al. (2013, 2014b), except that each stimulus was presented six times: three times with a letter that was present in it, and three times with a letter that was not. All 78 words were used in the incidental learning phase, resulting in a total of 468 trials. The trials were split into six blocks of 78 trials, with each word appearing once in each block. For the stimuli that included auditory FL word forms only, participants saw a series of hash symbols (#####) instead of the written NL translation that appeared in the other two word conditions (see Figure 1). Participants were instructed to respond “no” for these trials—that is, the letter was not contained in the written word, as no written word was presented. All written stimuli were displayed using Courier New font, size 20, in bold letters. Importantly, the written word forms were presented in the NL of the participants (English), unlike the experiments reported in Bisson et al. (2013, 2014b).

The session started with the set-up and calibration of the eye-tracker using a 9-point calibration grid. Following this, a series of eight practice trials with feedback preceded the start of the main experiment. Each trial in the experiment started with the presentation of the to-be-searched letter at the top of the screen for 500 ms. Then the picture or a blank screen was displayed in the middle of the screen for 300 ms before the presentation of the written word (or hash symbols) at the bottom of the screen. The auditory FL word onset was simultaneous with the onset of the written target string (word or hash symbols). The picture and written word stayed on screen until the end of the trial. The termination of each trial occurred when participants made a response, unless the auditory FL word was still playing. In those cases, the trial ended with the offset of the auditory FL word. A short break was included after each block allowing for the recalibration of the eye-tracker. The stimuli were displayed using SR Research's Experiment Builder software.

##### Phase 2: Explicit learning

Both groups of participants completed the explicit learning phase in which they were asked to complete a translation recognition task. The procedure for this phase was identical to that for the explicit learning phase used in Bisson et al. (2013, 2014b). For each trial, participants were presented simultaneously with a FL auditory word form and a possible NL written translation. Their task was to indicate with a button press whether the written NL word was the correct translation of the FL auditory word form. They received feedback on their answers (“correct” vs. “incorrect”), and they were asked to use this feedback to learn the correct FL–NL word pairs. Each FL word was presented once with its correct translation and once with a foil in each block of trials. The foils were selected from amongst all the NL translations, and they were different in each block. At the end of each block, the percentage accuracy was displayed on screen, and participants were reminded that their goal was to reach 80% correct in one block. If the criterion was reached, the explicit learning task was terminated, otherwise participants continued up to a maximum of three blocks of explicit learning.

##### Phase 3: Recall and translation recognition test

Participants in the experimental and control groups came back one week after Phase 2 to complete a recall and translation recognition test. In the recall test, participants were presented with the auditory FL words and were asked to type their English translations. Participants were encouraged to enter a translation even if they were not sure it was correct. They also had the option of simply pressing the “enter” key to proceed to the next trial without entering an answer. No feedback was provided during this recall test. The translation recognition test was the same one as used in Phase 2, except that no feedback was provided, and participants only completed one block of 156 trials (2 × 78: Each target word was presented once with its correct translation and once with a foil).

For both Phase 2 and Phase 3, E-Prime was used to present the stimuli and record the responses. Furthermore, in both tasks the ordering of the trials was randomized for each participant.

### Results

#### Phase 1: Incidental learning and eye tracking

The accuracy scores on the letter-search task were high (*M* = 98.5%, *SE* = 0.2%) indicating that participants attended to both the letter and the written word.

**Figure 2.**
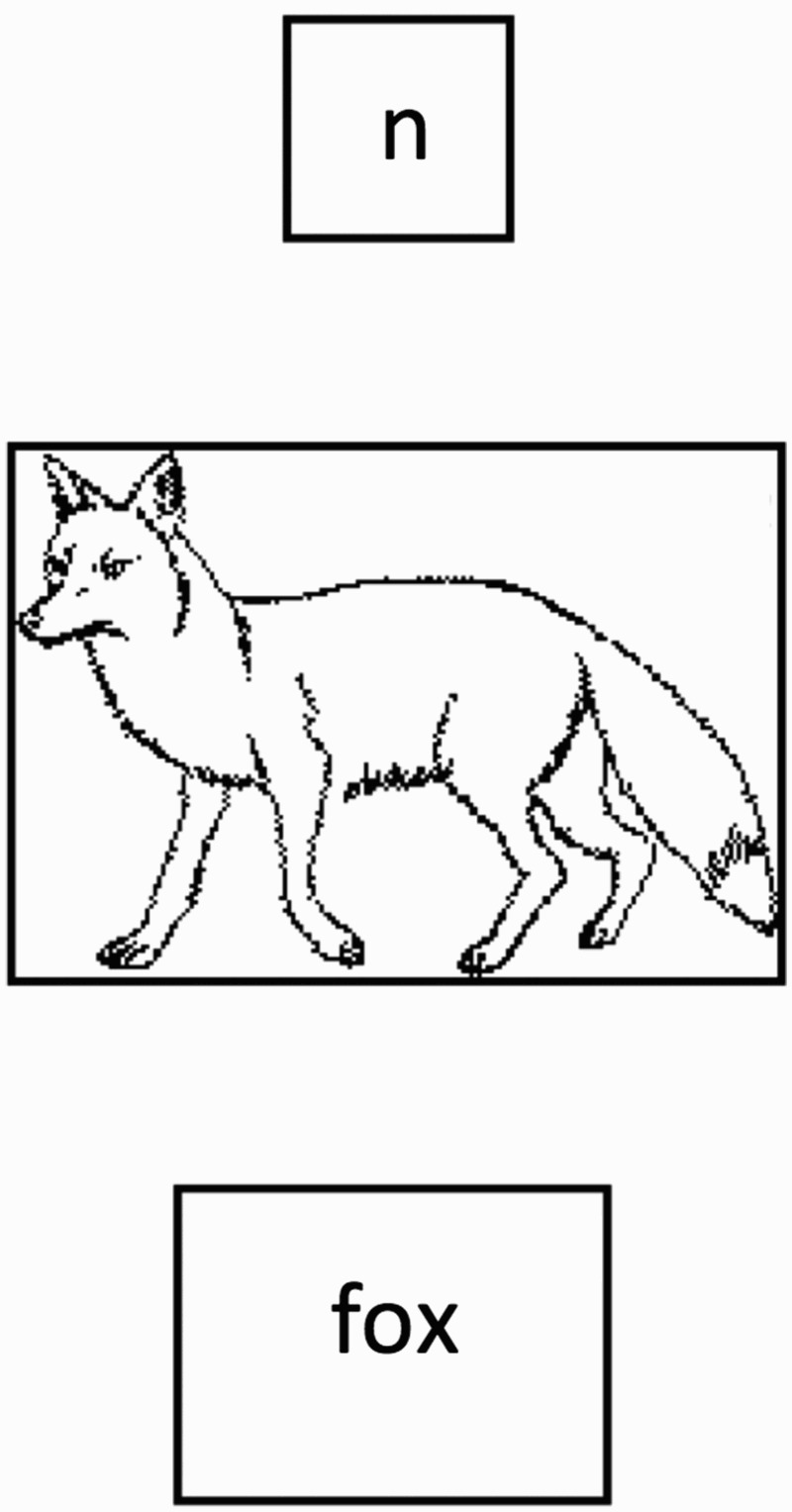
Example of a display screen showing the location of the letter area, the image area, and the word area. Fixations occurring elsewhere on the screen were defined as occurring in the “other” area (not actual size of stimuli).

For the purpose of the eye-tracking analyses, the screen display area was segmented into four regions of interest: the letter area, the image area, the word area, and the remaining display area (see Figure 2). Except the latter region, all regions of interest were centred along the *x*-axis. The letter area consisted of a region of 55 × 54 pixels starting 224 pixels from the top of the display area. The image area started on average 30 pixels below the letter area and varied in size according to the picture (average size 181 × 153 pixels). The word area started on average 97 pixels below the image area and also varied in size according to the length of the word (average size 137 × 76 pixels).

**Figure 3.**
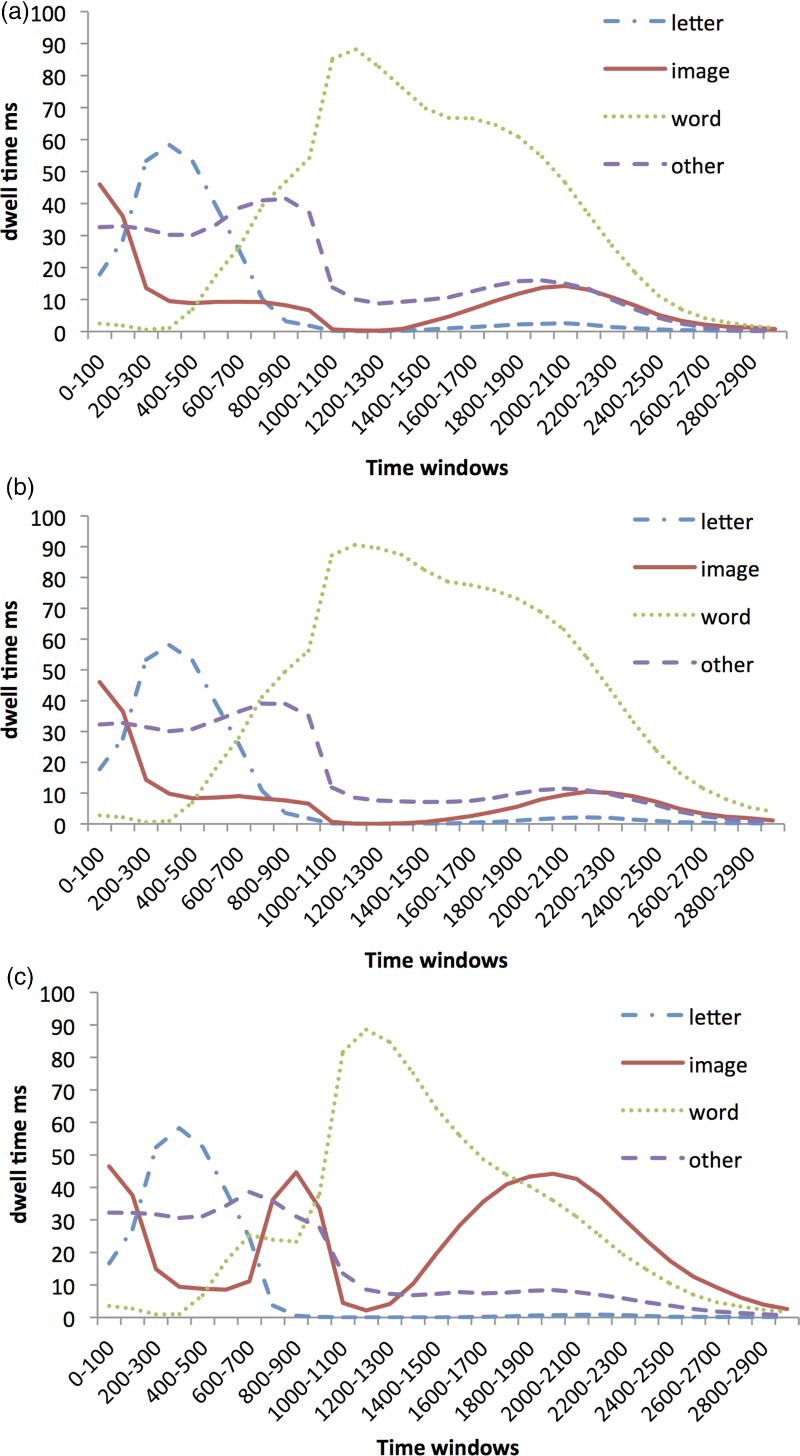
Dwell time in milliseconds (ms) in each region of interest in 100-ms time-windows from the onset of the letter for each word condition: (a) auditory word form only (A), (b) auditory and written word forms (AW), and (c) auditory word form and picture (AWP). The letter onset occurred at 0 ms and offset at 500 ms for all word conditions. The picture onset at 500 ms for the AWP condition. The word (hash symbols for the A condition) onset at 800 ms for all conditions. To view this figure in colour, please visit the online version of this Journal.

Table 1 shows the mean dwell time (total duration of all the fixations) in each region of interest averaged across participants and word conditions. Figure 3 shows the allocation of attention to the different regions of interest averaged across trials in time-windows of 100 ms for each word condition.

**Table 1.** Mean dwell time for each word condition and region of interest per trial

Area	Audio Dwell time (ms)	Audio–written Dwell time (ms)	Audio–written–picture Dwell time (ms)
Letter	278 (24)	275 (23)	252 (20)
Image	226 (30)	202 (26)	576 (46)
Word	974 (56)	1175 (54)	800 (56)
Other	452 (31)	417 (30)	385 (29)
All areas total	1931 (35)	2070 (34)	2014 (33)
Mean RT (ms)	634 (23)	773 (29)	717 (30)

*Note:* Response time (RT) is calculated from the moment the word appeared on the screen; dwell time is based on the whole trial duration. Standard errors in parentheses.

In order to investigate the allocation of attention to the different elements of the multimodal situation across the duration of the incidental learning task, the mean dwell times were calculated for each block and were submitted to repeated measures analysis of variance (ANOVA).

##### Dwell time in image area

**Figure 4.**
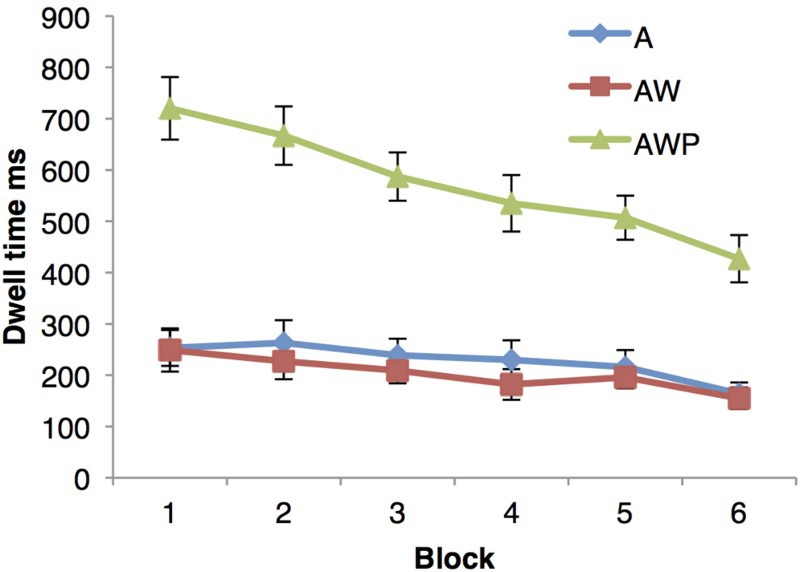
Dwell time in the image area in milliseconds (ms) across the incidental learning phase for each word condition with error bars (A = auditory FL only; AW = auditory FL with written NL translation; AWP = auditory FL with written NL translation and picture; FL = foreign language; NL = native language). To view this figure in colour, please visit the online version of this Journal.

As a picture was not presented in the condition with auditory FL and written information (AW) or auditory FL only (A; both these word conditions included a blank screen instead), the dwell time in the image area was only investigated in the condition with auditory, written, and pictorial information (AWP). The dwell time in the image area was investigated with block as a within-subject factor (6 levels: Blocks 1 to 6). Results revealed a significant linear trend, *F*(1, 27) = 32.59, *p* < .001, $\eta_{p}^{2}$ = .55, indicating that participants spent less time fixating in the image area as they progressed through the incidental learning task (Figure 4).

##### Dwell time in word area

**Figure 5.**
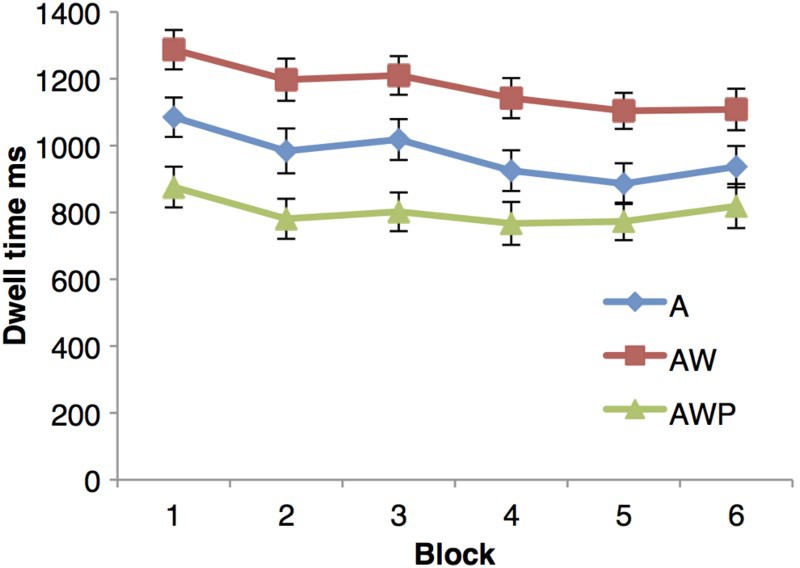
Dwell time in milliseconds (ms) in the word area across the incidental learning phase for each word condition with error bars (A = auditory FL only; AW = auditory FL with written NL translation; AWP = auditory FL with written NL translation and picture; FL = foreign language; NL = native language). To view this figure in colour, please visit the online version of this Journal.

For the word area, the mean dwell times were submitted to a 6 × 3 repeated measures ANOVA with block number (6 levels: Block 1 to Block 6) and word condition (3 levels: A, AW, AWP) as within-subject factors. The results revealed a main effect of block, *F*(3.20, 86.48) = 5.80, *p* < .01, $\eta_{p}^{2}$ = .18, and a main effect of word condition, *F*(1.37, 36.99) = 72.58, *p* < .001, $\eta_{p}^{2}$ = .73, as well as a significant interaction, *F*(5.16, 139.37) = 3.06, *p* < .05, $\eta_{p}^{2}$ = .10. Pairwise comparisons (Bonferroni corrected) were conducted to investigate the main effect of word condition further. These revealed that participants spent significantly longer looking in the word area in the condition with auditory FL and written NL translation than in both the condition with auditory FL word form only (where a series of hash symbols was presented) and the condition with auditory FL, written translation, and picture, all *p*s < .001 (see Table 1). Interestingly, participants spent longer fixating on the hash symbols in the auditory FL condition than on the word in the condition with auditory FL, written translation, and picture, *p* < .001. In other words, participants spent the least time looking in the word area when a picture was presented. To break down the interaction between word condition and block, separate repeated measures ANOVAs were computed for each word condition. These revealed significant linear trends for both the condition with auditory FL and written translations and the condition with auditory FL only [*F*(1, 27) = 14.15, *p* < .01, $\eta_{p}^{2}$ = .34; *F*(1, 27) = 13.24, *p* < .01, $\eta_{p}^{2}$ = .33, respectively], indicating that participants spent less time looking at the word area for both conditions as they progressed through the incidental learning task. However, for the condition that also included a picture (AWP), there was a significant quadratic trend, *F*(1, 27) = 9.75, *p* < .01, $\eta_{p}^{2}$ = .27 (see Figure 5) suggesting that the dwell time in the word area decreased between the first two blocks, then plateaued, and increased again during the latter blocks.

As can be seen from Figure 3, the addition of the picture seemed to change the allocation of attention during trials. First, shortly following the onset of the picture, there was a peak in the time spent looking in the image area (around 800 ms from the beginning of the trials). The second peak occurs at around 1900 ms, presumably following the completion of the letter-search task. To confirm this, the dwell times in the word area were submitted to repeated measures ANOVAs for the 800–900-ms and 1900–2000-ms time-windows with word condition as a within-subject factor. Results revealed significant main effects of word conditions for both time-windows [*F*(1.19, 32.21) = 85.19, *p* < .001, $\eta_{p}^{2}$ = .76; *F*(1.45, 39.20) = 62.27, *p* < .001, $\eta_{p}^{2}$ = .70, for 800–900 ms and 1900–2000 ms, respectively]. Contrasts revealed that participants spent the least time looking in the word area in the condition with pictorial information compared to both the condition with auditory FL word only [*F*(1, 27) = 75.79, *p* < .001, $\eta_{p}^{2}$ = .74; *F*(1, 27) = 33.62, *p* < .001, $\eta_{p}^{2}$ = .56] and the condition with auditory FL word form and written word [*F*(1, 27) = 106.85, *p* < .001, $\eta_{p}^{2}$ = .80; *F*(1, 27) = 87.30, *p* < .001, $\eta_{p}^{2}$ = .76] for both 800–900-ms and 1900–2000-ms time-windows, respectively (see Figure 3).

##### Dwell time in letter area

The mean dwell times in the letter area were also submitted to a 6 × 3 repeated measures ANOVA with the same factors as above. The results revealed a main effect of condition, *F*(2, 52) = 13.04, *p* < .001, $\eta_{p}^{2}$ = .33; however, neither the main effect of block nor the interaction between word condition and block was significant [*F*(5, 135) = 1.67, *p* = .33, $\eta_{p}^{2}$ = .04; and *F* < 1, respectively]. To investigate the main effect of condition further, pairwise comparisons (Bonferroni corrected) were conducted. These revealed that participants spent longer fixating in the letter area for both the condition with auditory FL word only and the conditions with auditory FL word with written translation than for the condition that also included a picture (AWP), *p* < .001 and *p* < .01, respectively (see Table 1). However, there was no significant difference between both conditions that did not include a picture (A vs. AW, *p* = 1).

##### Response time

**Figure 6.**
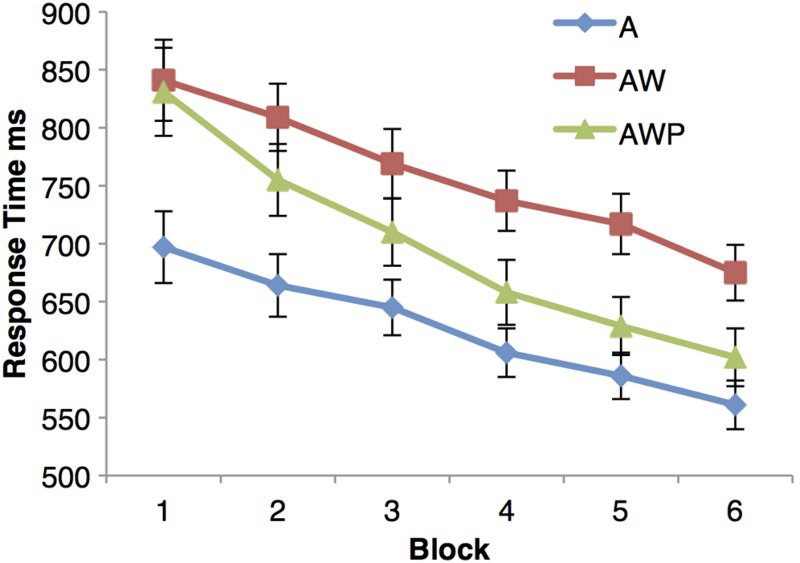
Response time in milliseconds (ms) for each word condition with error bars (A = auditory FL only; AW = auditory FL with written NL translation; AWP = auditory FL with written NL translation and picture; FL = foreign language; NL = native language). To view this figure in colour, please visit the online version of this Journal.

Incorrect responses (1.5% of data) and outliers (responses faster than 250 ms and slower than 2000 ms, 1% of data) were removed before the analysis. The mean response time was calculated for each participant and word condition and was averaged for each block.^1^1Response times in the analyses by item were log transformed to reduce kurtosis. These were submitted to a 6 × 3 repeated measures ANOVA with the same factors and levels as above. The results revealed a main effect of block [*F*_1_(2.31, 62.37) = 37.22, *p* < .001, $\eta_{p}^{2}$ = .58; *F_2_*(5, 385) = 153.52, *p* < .001, $\eta_{p}^{2}$ = .67], with a significant linear trend [*F*_1_(1, 27) = 65.52, *p* < .001, $\eta_{p}^{2}$ = .71; *F*_2_(1, 77) = 695.99, *p* < .001, $\eta_{p}^{2}$ = .90], indicating that participants responded faster as they progressed through the incidental learning phase. In addition, results revealed a main effect of word condition [*F*_1_(1.65, 44.45) = 95.13, *p* < .001, $\eta_{p}^{2}$ = .78; *F*_2_(2, 154) = 211.79, *p* < .001, $\eta_{p}^{2}$ = .73]. Pairwise comparisons (Bonferroni corrected) revealed that participants responded faster when the trials included auditorily FL only (A) than when they included auditory FL, written translation, and pictorial information (AWP), but they responded faster in the latter case than when words were presented with auditory FL and written translation (AW), all *p*s < .001 (see Table 1). Finally, there was a significant interaction between block and word condition [*F*_1_(10, 270) = 7.70, *p* < .001, $\eta_{p}^{2}$ = .22; *F*_2_(10, 770) = 5.87, *p* < .001, $\eta_{p}^{2}$ = .07]. The interaction seems due to response times in Block 1 not being significantly different for the words presented with auditory and written information (AW) and the words presented with auditory, written, and pictorial information (AWP), *t*s < 1 (see Figure 6). Response times in all other blocks are significantly different between all three word conditions, all *p*s < .05, except in Block 6 for the comparison of words presented auditorily only (A) and those presented with auditory, written, and pictorial information (AWP) in the analysis by item, which showed a strong trend, *p* = .057.

#### Phase 2: Explicit learning

The mean accuracy scores were calculated for each participant for each block and each word type and were averaged for each group. As many participants had reached criterion in Block 2 and therefore did not proceed to Block 3, the results of Block 3 were not analysed.

For Blocks 1 and 2, the mean accuracy scores were submitted to a mixed-design ANOVA with group as a between-subjects factor (2 levels: control and experimental groups) and word condition in the incidental learning phase as a within-subject factor (3 levels, auditory FL word only, A; auditory FL word with written translation, AW; and auditory FL word with written translation and picture, AWP). For the control group, the within-subject factor of word condition was still used even though this group of participants did not take part in the incidental learning phase (participants in the control groups were assigned to one of the three lists of stimuli as per participants in the experimental group prior to taking part in the experiment; see Stimuli section).

**Figure 7.**
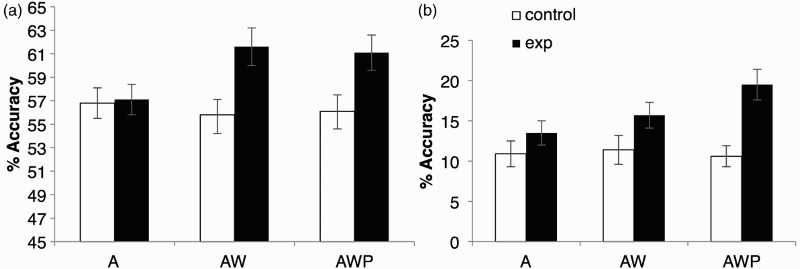
Percentage accuracy in (a) Block 1 of the translation recognition task (Phase 2) and (b) the recall task (Phase 3) for each group and word condition with error bars (A = auditory FL only; AW = auditory FL with written NL translation; AWP = auditory FL with written NL translation and picture; FL = foreign language; NL = native language; exp = experimental group).

For Block 1, the analysis revealed a main effect of group [*F*_1_(1, 51) = 9.22, *p* < .01, $\eta_{p}^{2}$ = .15; *F*_2_(1, 77) = 12.11, *p* < .01, $\eta_{p}^{2}$ = .14], indicating that the experimental group was overall more accurate than the control group (*M* = 59.9%, *SE* = 0.8%, and *M* = 56.3%, *SE* = 0.9%, respectively). The main effect of word condition was not significant [*F*_1_(2, 102) = 1.03, *p* = .36, $\eta_{p}^{2}$ = .02; *F*_2_ < 1]; however, there was a trend for an interaction between word condition and group [*F*_1_(2, 102) = 2.40, *p* = .096, $\eta_{p}^{2}$ = .05; *F*_2_(2, 154) = 2.53, *p* = .08, $\eta_{p}^{2}$ = .03] (see Figure 7). Simple effects analysis revealed that this was due to a significant effect of word condition in the experimental group [*F*_1_(2, 102) = 3.49, *p* < .05, $\eta_{p}^{2}$ =.07; *F*_2_(2, 76) = 3.01, *p* = .05, $\eta_{p}^{2}$ =.07], and not in the control group, *F*s < 1. Furthermore, simple effects analysis revealed that participants in the experimental group were more accurate on the words presented with auditory FL and written translation (AW) than those in the control group [*F*_1_(1, 51) = 7.58, *p* < .01, $\eta_{p}^{2}$ = .13; *F*_2_(1, 77) = 9.03, *p* < .01, $\eta_{p}^{2}$ = .11]. The same result was found for the words presented with auditory FL, written translation, and pictorial information (AWP) [*F*_1_(1, 51) = 5.95, *p* < .05, $\eta_{p}^{2}$ = .10; *F*_2_(1, 77) = 10.17, *p* < .01, $\eta_{p}^{2}$ = .12], but not for the words presented auditorily only (A), *F*s < 1.

As only the experimental group had been presented with the three word conditions, an effect of word condition was only expected in the experimental group. Hence the percentages of accuracy in the translation recognition task for the experimental group were submitted to a repeated measures ANOVA. The results showed a trend for a main effect of word condition [*F*_1_(2, 54) = 2.94, *p* = .06, $\eta_{p}^{2}$ = .10; *F*_2_(2, 154) = 2.86, *p* = .06, $\eta_{p}^{2}$ = .04]. Importantly, contrasts revealed that participants performed significantly better on the words presented with auditory FL and written translation (AW) than on the words presented with auditory FL only (A) [*F*_1_(1, 27) = 5.26, *p* < .05, $\eta_{p}^{2}$ = .16; *F*_2_(1, 77) = 4.93, *p* < .05, $\eta_{p}^{2}$ = .06]. Furthermore, there was a strong trend for participants to perform better on the words presented with audio, written, and pictorial information (AWP) than auditorily only (A) [*F*_1_(1, 27) = 4.11, *p* = .05, $\eta_{p}^{2}$ = .13; *F*_2_(1, 77) = 3.89, *p* = .05, $\eta_{p}^{2}$ = .05]. However, whether participants were also presented with the picture did not have any additional impact on their performance on the translation recognition task (AW vs. AWP, *F*s < 1).

To summarize the findings of Block 1, an incidental learning effect was found as the experimental group outperformed the control group overall. Further analyses revealed that this was due to the experimental group outperforming the control group for the words presented with meaning during the incidental learning phase (AW and AWP words). However, the additional pictorial information did not influence performance on the translation recognition task, as there was no significant difference between the words presented with audio, written, and pictorial information (AWP) compared to words presented with audio and written information only (AW).

The analysis of Block 2 also revealed a main effect of group that was significant by item, and a trend by participant [*F*_1_(1, 51) = 2.90, *p* = .095, $\eta_{p}^{2}$ = .05; *F*_2_(1, 77) = 13.98, *p* < .001, $\eta_{p}^{2}$ = .15] (*M* = 70.9%, *SE* = 1.4%, and *M* = 67.5% *SE* = 1.4%, for the experimental and control group, respectively). Neither the main effect of word condition [*F*_1_(2, 102) = 1.22, *p* = .30, $\eta_{p}^{2}$ = .02; *F*_2_(2, 154) = 1.74, *p* = .18, $\eta_{p}^{2}$ = .02], nor the interaction between group and word condition was significant, *F*s < 1.

#### Phase 3: Recall and translation recognition

##### Recall

The percentages of accuracy on the recall task were calculated for each participant in each group for each word condition. These were analysed by participant only, because many items were not recalled by any participants (accuracy = 0%). The percentages of accuracy were submitted to a mixed-design ANOVA with group as a between-subjects factor (2 levels: control and experimental groups) and word condition in the incidental learning phase as a within-subject factor (3 levels, A, AW, AWP). Results revealed a significant main effect of group, *F*(1, 51) = 8.81, *p* < .001, $\eta_{p}^{2}$ = .15, as well as a trend for a main effect of word condition, *F*(2, 102) = 2.60, *p* = .08, $\eta_{p}^{2}$ = .05. Importantly, the interaction between group and word condition was significant, *F*(2, 102) = 3.40, *p* < .05, $\eta_{p}^{2}$ = .06 (see Figure 7). This interaction occurred because the effect of word condition was significant in the experimental group, but not in the control group [*F*(2, 102) = 6.26, *p* < .01, $\eta_{p}^{2}$ = .12, and *F* < 1, respectively]. Simple effects analysis revealed that participants in the experimental group outperformed participants in the control group for the words presented originally with auditory, written, and pictorial information (AWP), *F*(1, 51) = 14.72, *p* < .001, $\eta_{p}^{2}$ = .29, and there was also a trend for the experimental group to perform better than the control group for the words presented originally with auditory and written information (AW), *F*(1, 51) = 3.32, *p* = .07, $\eta_{p}^{2}$ = .07. There were no significant differences between the groups for the words presented auditorily only (A) during the incidental learning phase, *F*(1, 51) = 1.25, *p* = .25, $\eta_{p}^{2}$ = .03.

In order to explore the effect of exposure to the different word conditions further, a repeated measures ANOVA was computed for the experimental group only. This revealed a significant main effect of word condition, *F*(2, 54) = 5.44, *p* < .01, $\eta_{p}^{2}$ = .17, as well as a significant linear contrast, *F*(1, 27) = 9.99, *p* < .01, $\eta_{p}^{2}$ = .27. Contrasts revealed that participants performed significantly better on the recall task if FL words had been presented auditorily with written translation and pictorial information (AWP) in the incidental learning phase than if they had been presented auditorily with written translation only (AW), *F*(1, 27) = 4.40, *p* < .05, $\eta_{p}^{2}$ = .14, or auditorily only (A), *F*(1, 27) = 9.99, *p* < .01, $\eta_{p}^{2}$ = .27. However there was no significant difference between the FL words presented auditorily with written translation (AW) and auditorily only (A), *F*(1, 27) = 91.46, *p* = .24, $\eta_{p}^{2}$ = .05.

##### Recognition

The mean percentages of accuracy on the recognition test were calculated for each participant in each group for each word condition and were submitted to a mixed ANOVA with group as a between-subjects factor (2 levels: control and experimental groups) and word condition in the incidental learning phase as a within-subject factor (3 levels: A, AW, AWP). Results revealed a significant main effect of group by items, *F*_2_(1, 77) = 4.17, *p* < .05, $\eta_{p}^{2}$ = .05, but not by participants, *F*_1_ < 1 (*M* = 73.7%, *SE* = 1.4%, and *M* = 71.8%, *SE* = 1.5%, respectively). The main effect of word condition and the interaction between group and word condition were not significant [*F*_1_(2, 102) = 1.18, *p* = .31, $\eta_{p}^{2}$ = .02; *F*_2_(2, 154) = 1.21, *p* = .30; and *F*s < 1, respectively.

#### Relationship between dwell time and learning

The impact of the dwell time in the image and word areas on the learning of the FL words presented auditorily with written translation (AW), as well as auditorily with written translation and pictorial information (AWP), was investigated using a generalized linear model with a logit. Each word condition was investigated separately.

##### Word condition: AWP

For the FL words presented auditorily with written translation and pictorial information (AWP) during the incidental learning phase, both the dwell time in the image area and that in the word area were investigated as potential predictors of the probability of obtaining a correct answer on the recall and recognition measures. The results showed that the dwell time in the image area was a significant predictor of the probability of obtaining a correct answer on both recall and recognition tests in Phase 3, and it was a strong trend for the recognition scores in Phase 2 for the second block of explicit learning (see Table 2). However, dwell time in the image area was not a significant predictor for the scores in Block 1 of the explicit learning phase, *p* = .66. Furthermore, adding the dwell time in the word area as a predictor did not significantly improve any of the models, *p*s > .61.

**Table 2.** Predictors of learning for FL words presented with written translations and pictures

							Pseudo R^2^		
Phase	Learning outcome	Predictors	B (SE)	OR	95% CI	χ^2^	HL	CS	N
Phase 2	Recognition Block 2					3.44^†^	.06	.12	.13
		Constant	0.63 (0.15)						
		DT picture	0.45 (0.24)^†^	1.57	[0.98, 2.53]				
Phase 3	Recall					4.47^*^	.09	.15	.18
		Constant	−1.91 (0.26)						
		DT picture	0.83 (0.39)^*^	2.29	[1.06, 4.96]				
Phase 3	Recognition					7.34^**^	.16	.23	.29
		Constant	0.65 (0.15)						
		DT picture	0.68 (0.25)^**^	1.98	[1.21, 3.26]				

*Note:* FL = foreign language; AWP = auditory–written–picture; DT = dwell time; *OR* = odds ratio; CI = confidence interval; HL = Hosmer–Lemeshow; CS = Cox–Snell; N = Nagelkerke. The values of pseudo *R*^2^ for the Phase 3 recall model change to .13, .15, .23, respectively, once the two participants with 0% correct recall are removed.

† *p* = .06.

* *p* < .05.

** *p* < .01.

As was mentioned earlier, there were two peaks in the time spent looking at the picture during each trial (see Figure 3c). As this was the case, the dwell time on the picture was split into two time-windows to capture the early and the later time spent looking at the picture during each trial. The early time-window included any dwell time between 600 and 1100 ms post trial onset (first peak in Figure 3c), and the later time-window included the dwell time between 1200 and 3000 ms (second peak in Figure 3c). These two time-windows were then used in the logistic regression to predict learning outcomes. The results showed that the dwell time on the picture during the later time-window was a significant predictor of the probability of obtaining a correct answer on the recall and recognition test during Phase 3 as well as in Block 2 of the explicit learning task (Phase 2; see Table 3). However, the dwell time in the image area during the early time-window was not a significant predictor, all *p*s > .56.

**Table 3.** Predictors of learning for FL words presented with written translations and pictures (AWP) during 1200–3000 ms time-window

							Pseudo R^2^		
Phase	Learning outcome	Predictors	B (SE)	OR	95% CI	χ^2^	HL	CS	N
Phase 2	Recognition Block 2					5.48^*^	.10	.18	.20
		Constant	0.63 (0.12)						
		DT picture	0.75 (0.32)^*^	2.11	[1.13, 3.96]				
Phase 3	Recall					5.78^*^	.11	.19	.22
		Constant	−1.87 (0.22)						
		DT picture	1.25 (0.52) ^*^	3.47	[1.26, 9.71]				
Phase 3	Recognition					9.14^**^	.20	.28	.35
		Constant	0.70 (0.13)						
		DT picture	1.00 (0.33)^**^	2.72	[1.42, 5.24]				

*Note:* FL = foreign language; AWP = auditory–written–picture; DT = dwell time; *OR* = odds ratio; CI = confidence interval; HL = Hosmer–Lemeshow; CS = Cox–Snell; N = Nagelkerke. The values of pseudo *R*^2^ for the Phase 3 recall model change to .16, .15, .24, respectively, once the two participants with 0% correct recall are removed.

* *p* < .05.

** *p* < .01.

##### Word condition: AW

For the FL words presented auditorily with written translation (AW), analyses revealed that the dwell time on the word was not a significant predictor of learning, neither in Phase 2 nor in Phase 3, all *p*s > .31.

## General Discussion

One of the aims of this study was to investigate the allocation of attention to the different elements of the multimodal situation. The eye-tracking data confirmed that both the letter and the written information were processed in all word conditions. However, the time spent looking in the word area differed across conditions. When only the written NL translation was presented on screen, participants spent about half of the trial duration fixating in the word area. However, this decreased significantly when the word condition also included pictorial information: Participants spent less time looking in the word area when a picture was presented first. Furthermore, response times were faster in the condition that included a picture than in the condition with written NL only. Because the picture onset occurred 300 ms prior to the onset of the written word, the results suggest that the processing of the picture primed the NL orthographic word form. In other words, the processing of the pictorial information activated semantic representations and lexical representations. When participants then searched the word to complete the letter-search task, the preactivated lexical representations would have facilitated the processing of the written word forms. Finally, the results showed that participants returned to the image area once the letter-search task had been completed.

The second reason for using eye tracking was to investigate the allocation of attention across the duration of the incidental learning phase. Based on the results of Bisson et al. (2014b), it was expected that more attention would be allocated to the pictorial stimuli at the beginning of the incidental learning phase because of its novelty. Furthermore, since the pictures were irrelevant for the letter-search task, it was expected that as the experiment progressed, and the pictures became less salient because they had already been viewed, less time would be devoted to looking at them. The results confirmed this prediction, as the time spent fixating in the image area decreased significantly across blocks of trials. In fact, the dwell time in the image area for the condition including a picture, decreased by about half between the beginning and the end of the incidental learning task, suggesting that the “irrelevant pictorial information” did lose some of its appeal. Participants also spent less time looking in the word area for both conditions with auditory and written information, and auditory information only, presumably because they could solve the letter-search task faster as the experiment progressed, due to the repetition of the words.

In the present experiment, FL input was only presented auditorily, and in order for the acquisition of FL vocabulary to occur, the FL auditory word forms had to be processed. Thus, if there is evidence of FL vocabulary acquisition through exposure to the multimodal incidental learning task, it can only be concluded that the FL auditory word forms were processed. The results of the explicit learning task completed one day following the incidental learning phase confirmed that exposure to the FL words led to the acquisition of FL vocabulary as participants in the experimental group achieved higher accuracy overall than participants in control group. Furthermore, an incidental learning effect was found in the recall task one week later, as again participants in the experiment group outperformed participants in the control group. Taken together, the results showed that the auditory FL words were processed, even though they were not relevant for the letter-search task.

Interestingly, being exposed to auditory FL word forms only was not enough to lead to an incidental learning advantage. This was surprising, especially considering that the letter-search task was potentially easier in this condition as participants were presented with a series of hash symbols (#####) rather than a written word, and therefore they could have allocated more cognitive resources to processing the auditory FL word forms. The important aspect that led to gains in vocabulary knowledge was the presence of both FL auditory word form and meaning information. However, having access to the meaning of the words through the NL translation only or both NL translation and pictorial information did not influence accuracy scores differently in the explicit learning task. However, in the recall task one week later, participants achieved significantly higher recall for the words presented with pictures and NL translations than for the words presented with NL translation only.

In contrast to the results of Bisson et al. (2013, 2014b), there was no incidental learning advantage in the second block of explicit learning, as the control group performed similarly to the experimental group. Furthermore, there were no significant differences between the groups in the recognition task completed one week later. Therefore, it seems that through the explicit learning of translation equivalents, the control group quickly caught up with the experimental group.

The last aim of the study was to assess the impact of the pictorial information on incidental learning. The results showed that having pictorial information during the incidental learning phase was beneficial for recall one week later. Whether the pictorial information helps recall in general, or whether it is helpful only in the case of delayed recall, is unclear as the recall task was not completed until one week after the incidental learning phase. However, as the goal of language learning is to be able to use the acquired vocabulary after a time delay, the results showed that pictorial information can play an important part in incidental vocabulary learning. Furthermore, the advantage gained from the pictures during the incidental learning phase occurred after only six presentations of the pictorial information, even though the pictures were irrelevant for the task, and the meaning of the FL words could already be accessed through the written translations. The results therefore suggest a special role for pictorial information. This was supported by the finding that the dwell time on the pictures during the incidental learning phase was a significant predictor of recall scores, thereby highlighting the usefulness of pictorial information for vocabulary learning. Although results did not reveal an advantage of having been exposed to pictorial information on the delayed recognition test (possibly due to a ceiling effect), the dwell time on the picture was also a predictor of the recognition scores one week later. Furthermore, there was a strong trend for the dwell time on the picture to predict the recognition scores in the second block of explicit learning the day following the incidental learning phase. Importantly, it was found that it was the dwell time on the picture in the later time-window that significantly impacted learning. It may be that looking at the picture during the early time-window (prior to the onset of the written word) did not help learning because participants were focused on the letter-search task—that is, as they started processing the picture, the onset of the written word directed their attention to the letter-search task. In the later time-window, however, as participants had already solved the letter-search task, they had more cognitive resources available to benefit from the exposure to the picture. It is also possible that it is the processing of the picture following the processing of the written word that impacted learning. In other words, by the time participants processed the picture in the later time-window, they already had a quick preview of the pictorial information, *and* they had processed the written label. Thus, the combination of this information may have boosted the impact of the pictorial information during the latter time-window. Crucially, one important difference between the early and later time-windows is the onset of the auditory FL word. Seeing the pictures prior to hearing the FL word (as in the early time-window) did not impact learning, whereas processing the picture following the onset of the auditory FL word (as in the later time-window) did. This indicates that the timing of the presentation of the different elements of the multimodal situation has important implications for learning.

Another important consideration that may explain the different pattern of results between the FL words presented with pictorial information (AWP) compared to those with NL translations only (AW) is the type of memory encoding resulting from these two types of words presentation. As participants only had to process the NL translations visually in the AW condition to complete the letter-search task, semantic access was not required. Thus in this condition, FL words may simply have formed connections to NL word forms (i.e., lexical connections). However, in the AWP condition, the appearance of the picture is likely to have triggered activation of semantic representations. This in turn may have helped to establish connections between FL word forms and semantic representations (i.e., lexical–semantic connections). The results of the translation recognition task performed the day after the incidental learning task revealed no difference between the AW and AWP conditions. This suggests that after one day, both lexical connections and lexical–semantic connections were sufficiently strong to allow participants to perform the task. In contrast, as participants performed better on the AWP words in the recall task after a week delay, this could be taken as evidence that only lexical–semantic connections remained sufficiently strong, allowing participants to perform the task. Although further research is needed to confirm this, the different pattern of result does suggest different types of connections, with the processing of pictorial information leading to stronger connections.

How could pictures support vocabulary learning? In memory research, superior recall for pictures has generally been found. Dual-coding theory states that this is because pictorial information can be encoded in memory both as nonverbal information and as verbal information, by generating a lexical label (Paivio & Csapo, 1973). The cascading activation model of speech production supports the idea of automatic activation of lexical information during picture processing, even when the pictures are irrelevant for a task (see Kuipers & La Heij, 2009; Meyer & Damian 2007; Morsella & Miozzo, 2002; Navarrete & Costa, 2005). In the present experiment, both pictorial and written NL information were available during the incidental learning phase; therefore the encoding of both types of information would have been encouraged, and it is not possible to evaluate the impact of the picture alone. It is therefore possible that it was the combination of written and pictorial information that was beneficial. However, the time spent looking in the word area was not a predictor of learning, and therefore this suggests that the picture played a crucial role. Importantly, is the processing of the picture, notwithstanding the written label, beneficial for vocabulary acquisition? Of course, we cannot determine what kind of cognitive processes occurred whilst participants looked at the picture (i.e., were they processing the visual aspect of the picture, the semantic information it contained, and/or activating the verbal label for the picture?). However, it is unlikely that the performance on the one-week delay recall task can simply be attributed to the activation of the NL word representation whilst processing the pictures; thus it must be the combination of information provided by the picture that is crucial. The semantic–sensory model posits that access to semantic representations is faster and more direct for pictures than for words, and that as pictures are more distinctive and varied in their mnemonic features they are more easily recalled (Nelson et al., 1977). Although the results of the current experiment do not allow us to pinpoint why FL words learnt in combination with pictures are better recalled than FL words learnt in combination with NL words (nor was it the aim), both explanations—that is, dual coding and direct semantic access—are plausible. Furthermore, as was suggested earlier, it may well be that having access to pictorial information during encoding promotes the creation of direct connections between the FL words and the semantic representations, which facilitates recall later on. Crucially, what the results suggest is that the pictures were a richer source of information than the written words and promoted a deeper processing, which was more beneficial for learning.

The results of the current study are in contrast to both Lotto and de Groot (1998) and Carpenter and Olson (2011), as our data revealed an advantage for learning FL words in combination with pictures. In spite of the FL learning context of these two studies, an important factor that may have contributed to the lack of picture superiority effect is the learning paradigm used. The previous studies involved explicit learning, whereas the current study used an incidental learning paradigm. Importantly, the benefit of having access to pictures in the current study emerged one week following the learning phase, and neither of the two studies mentioned above included a delayed test. Therefore, the results found here are important in showing that in contrast to what has been found in previous studies of FL word learning, there is a benefit of having access to pictures for FL vocabulary learning. It remains to be seen whether this is the case only for incidental learning paradigms, and whether the learning benefits of pictorial information need more time to emerge. In addition, as the advantage for having access to pictorial information in the current study emerged one week following the incidental learning phase, and participants also completed an explicit learning phase in between, it is possible that the advantage emerged through having been exposed to both types of learning—that is, incidental and explicit. In other words, it is unclear at the moment whether the advantage was due to incidental learning alone or to the combination of initial incidental learning followed by explicit learning. Although the pictorial information was only provided during the incidental learning phase, it is possible that the explicit learning phase had a differential impact on the words presented initially with auditory, written, and pictorial information compared to auditory and written information only.

The results of the current study revealed an important role for pictorial information in FL vocabulary learning. Not only did the pictures alter the viewing behaviour during the incidental learning phase, but they also helped participants retrieve the correct translations of FL words one week later. This highlights the importance of using delayed testing before concluding on the usefulness of a training method in FL teaching and learning. The FL words were presented auditorily in the current study, and combining this with both NL translations and pictures in an incidental learning situation was an effective method of learning FL vocabulary. It remains to be seen whether this combination of input modalities would also lead to FL vocabulary acquisition benefits for more complex multimodal situations like films with subtitles.
